# Simplifying Mitral Valve Repair with Novel Premeasured Chordal Loops

**DOI:** 10.3390/jcm13237029

**Published:** 2024-11-21

**Authors:** Daniel Shell, Natcha Bunwatcharaphan, Michael Seitz, Michael Rowland, Manoras Chengalath, Cheng-Hon Yap

**Affiliations:** 1 Department of Cardiothoracic Surgery, Barwon Health, Geelong, VIC 3220, Australia; natchabu@kku.ac.th (N.B.); manoras.m@gmail.com (M.C.); chenghonyap@gmail.com (C.-H.Y.); 2 School of Medicine, Deakin University, Geelong, VIC 3216, Australia; 3 Department of Epidemiology and Preventive Medicine, Monash University, Melbourne, VIC 3004, Australia

**Keywords:** cardiac surgery, valvular heart disease, mitral regurgitation, mitral valve repair, neochordae

## Abstract

**Background:** The ”respect” approach to surgical mitral valve repair, which involves implanting artificial neochordae, is gaining increased adoption. Surgeons are possibly prone to error in the manual construction of neochordae, which can lead to prolonged cross-clamp times. Novel systems such as Chord-X Pre-Measured Loops (On-X Life Technologies, Inc., Austin, TX, USA) eliminate the need for manual neochordae construction, potentially simplifying the mitral repair procedure. However, clinical data on its use are currently limited to a small publication. **Methods**: We conducted a retrospective cohort study to evaluate the use of Chord-X loops in 40 consecutive patients who underwent surgery in Geelong, Victoria, Australia, between May 2020 and February 2024. Three surgeons participated in this study. **Results**: All patients were referred for severe mitral valve regurgitation secondary to myxomatous degeneration, with P2 prolapse being the most common pathology. Chord-X Pre-Measured Loops effectively corrected a variety of leaflet pathologies, including bi-leaflet disease, with a single set of loops sufficing in most patients. Intraoperative and follow-up echocardiographic assessments revealed no greater than mild mitral regurgitation in any patient, with 75% exhibiting no or trace mitral regurgitation. **Conclusions**: The Chord-X Pre-Measured Loops system demonstrated safety, efficacy, and reproducibility across all patients. Surgeons were able to easily adopt this technology without requiring additional training. We believe this technology offers a safe option for surgeons performing low-volume mitral repair surgeries.

## 1. Introduction

The “respect” approach to mitral valve repair, which involves the implantation of artificial neochordae to constrain and correct excessive leaflet motion, is increasingly favoured over resecting abnormal leaflet tissue [1]. Emerging evidence indicates that this approach may result in improved haemodynamics and long-term survival [2]. However, the success of this method hinges on the precise fixation and sizing of the neochordae to ensure optimal tension and leaflet coaptation. Traditionally, surgeons have manually constructed these chords, a process that is potentially prone to error and can be time-consuming during the critical cross-clamp period.

Novel systems such as Chord-X Pre-Measured Loops (On-X Life Technologies, Inc., Austin, TX, USA) offer a simplified and reproducible method for using artificial chordae, eliminating the need for manual construction by the surgeon and facilitating straightforward implantation. To date, only one published series of 17 patients has reported on the use of this technique [3]. Our study aims to expand the knowledge base of this innovative mitral valve repair technology by documenting our experience and early repair outcomes in our first 40 patients.

## 2. Materials and Methods

### 2.1. Surgical Technique

The mitral valve and subvalvular apparatus are exposed using the surgeon’s preferred operative approach. The visual inspection identifies the diseased valve leaflets requiring neochordae support and the most suitable papillary muscle(s) for chordal loop attachment. The ideal chordal loop size is measured from the fibrous portion of the papillary muscle to an adjacent, un-diseased leaflet to achieve optimal leaflet coaptation. Measurements are made using the Chord-X Chordal Sizer or a disposable ruler. Once the appropriate size is selected, the corresponding number of Chord-X Pre-Measured Loops sets are introduced to the sterile field. These can be used immediately, requiring no further surgeon or scrub team preparation.

**Figure 1:**
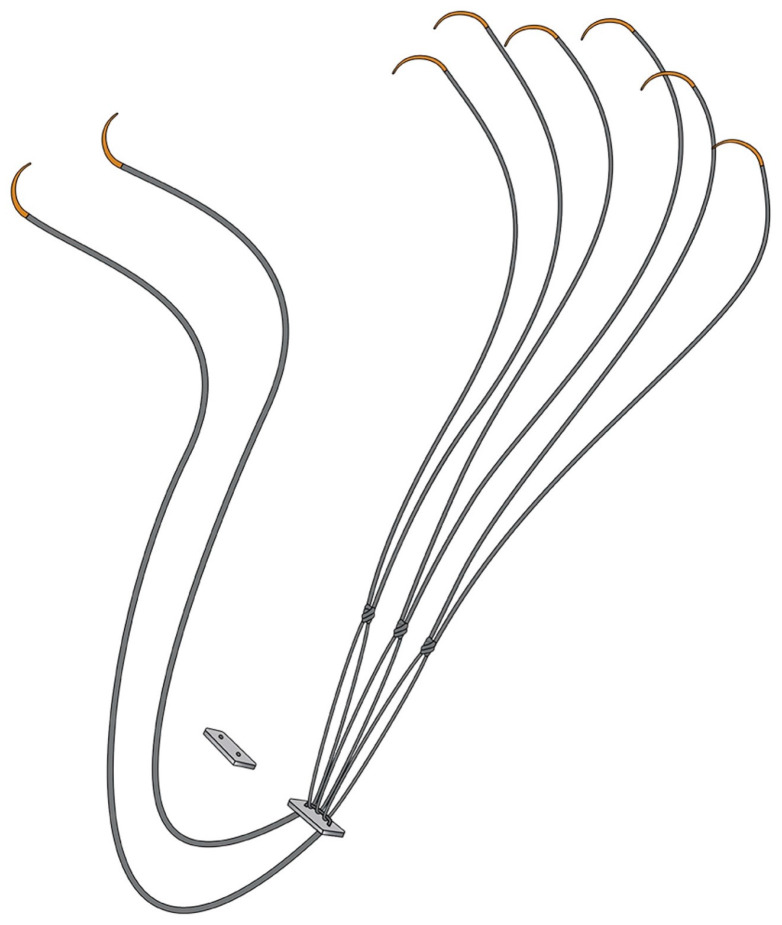
Diagram of Chord-X Pre-Measured Loops system demonstrating the central pledget and attached ePTFE sutures. Used with the permission of Artivion, Inc., Kennesaw, GA, USA.

**Figure 2:**
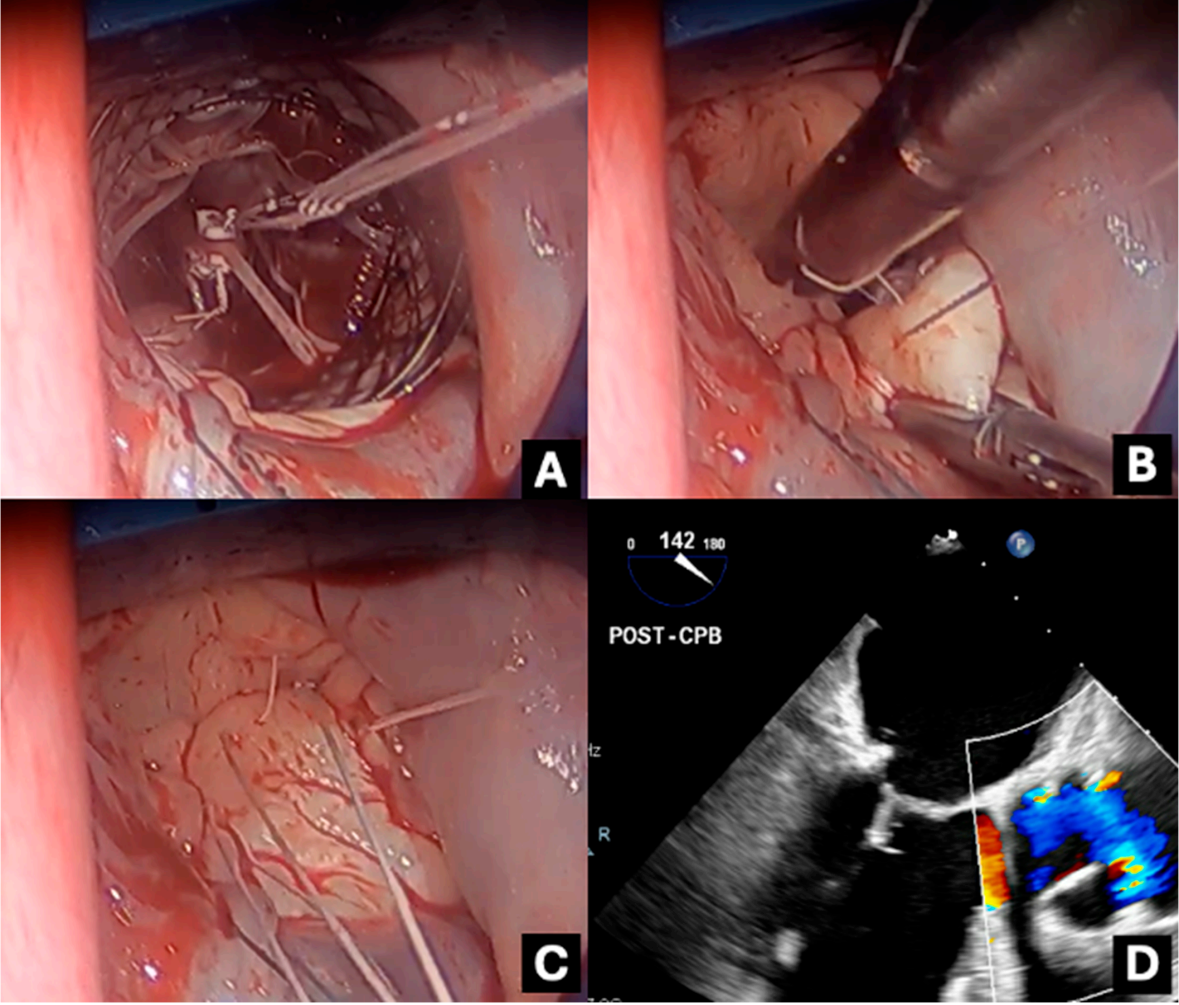
Intra-operative photography and TOE imaging demonstrating: (**A**) pledget fixation of the Chord-X system onto the posteromedial papillary muscle, (**B**) suturing of loops onto the P2 leaflet edge, (**C**) all loops attached to the P2 leaflet edge, and (**D**) mid-oesophageal long axis view post-repair exhibiting adequate coaptation of mitral valve; the neochord is faintly seen attaching to posterior aspect of the valve.

Each Chord-X Pre-Measured Loops set includes a central pledget with two free needles exiting from one side and three pre-configured chordal loops (with attached needles) emerging from the opposite side (Figure 1). The threads are composed of expanded polytetrafluoroethylene (ePTFE). The two free needles anchor to the fibrous portion of the papillary muscle and tie onto a second free pledget (Figure 2A). The chordal loops are subsequently sutured via their associated needles to the diseased mitral valve leaflets and tied (Figure 2B,C). The number of knots and tension in the knot does not change the neochordae size. Although each set has three loops, attaching all loops to the valve is not mandatory. Unused loops can be discarded without compromising the system’s integrity or strength. Post-cardiopulmonary bypass (CPB) trans-oesophageal echocardiography (TOE) is used to confirm the adequacy of repair (Figure 2D).

### 2.2. Study Design

We designed a retrospective observational cohort study to investigate the short-to-medium-term outcomes of using Chord-X Pre-Measured Loops. From May 2020 to February 2024, three surgeons in Geelong, Victoria, Australia, performed 40 consecutive mitral valve repair operations using Chord-X Pre-Measured Loops. Patients were selected for this operative technique based on pre-operative echocardiography, indicating that their mitral valve pathology was amenable to repair. Patients who underwent mitral valve repair during the study period without neochordae (e.g., annuloplasty only) were not included in this study.

All 40 patients with neochordae were included in this retrospective study and analysed. Data were collected retrospectively from the patient’s medical records, including operative notes, discharge paperwork, and post-operative echocardiography reports. The duration and frequency of follow-up echocardiography were determined by the patient’s treating cardiologist, and only data from the latest follow-up were evaluated. All data extraction, cleaning, and statistical analysis were performed using R version 4.3.0 (R Core Team, Vienna, Austria).

Barwon Health Human Research Ethics Committee (QA/104853/VICBH-2023-405717(v1), 13 December 2023) approved this study and granted a waiver of written consent due to the negligible risk of this research.

## 3. Results

All 40 patients were referred for surgery due to severe mitral regurgitation identified via transthoracic (TTE) echocardiography, with most patients undergoing a follow-up TOE to further clarify the mechanism of regurgitation. The underlying pathology was myxomatous valve degeneration in all cases, with no instances of rheumatic or ischemic mitral disease in this dataset (Table 1). All but three procedures were performed electively. Approximately one-third of patients were asymptomatic despite severe mitral valve disease, but these individuals exhibited evidence of early left ventricular dilatation and thus met the indications for surgical restoration (Table 2). Most patients had preserved left ventricular function, and the mean pulmonary artery pressure was within the normal range. Other than the single emergency case, these patients represented a low-risk surgical cohort (EuroSCORE II < 3%). Patient demographics are further detailed in Table 1.

The choice of operative approach was primarily influenced by favourable thoracic anatomy and the absence of combined procedures, with six patients undergoing a minimally invasive approach (via right lateral mini-thoracotomy). Nine patients underwent concomitant procedures (six CABG, one tricuspid valve repair, and two bi-atrial MAZE). Intraoperative inspection of the mitral valve confirmed P2 leaflet pathology in 85% of patients, with P3 involvement (concomitant or standalone) in 55% (Table 2). Although pre-operative echocardiography (either transthoracic or trans-oeosphageal) determined leaflet prolapse as the underlying mechanism in the majority of cases, many patients were found to have abnormal or ruptured chordae on intra-operative inspection. The anterior leaflet was implicated in only one patient in this series who had prolapse of the entire anterior complex; aside from requiring longer neochordae, no major modifications to the implantation technique were necessary.

**Figure 3:**
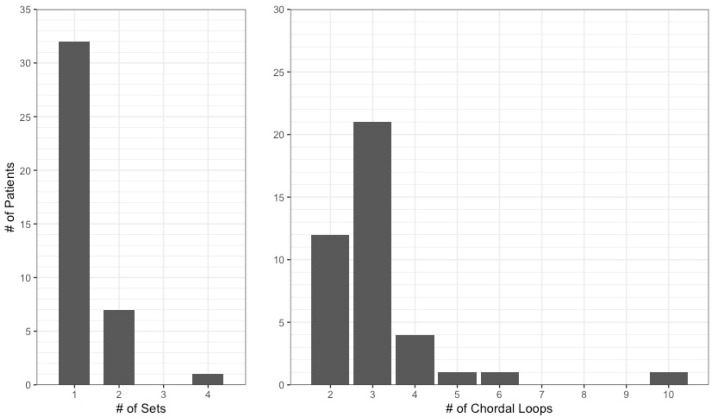
Distribution of the number of Chord-X sets and individual loops required per patient.

Most Chord-X loops were anchored to the posterior papillary muscle, with only 11 patients requiring chords attached to the anterior papillary muscle. In general, the papillary muscle attachment was chosen so that neochordae do not cross the mitral valve ‘midline’ and thus respect native chordal arrangements [4,5]. For disease of the posterior mitral leaflets, 16 mm pre-measured loops were the most appropriate size for 85% of patients, with the remainder requiring 12 mm loops (Table 3). In the single patient with bi-leaflet prolapse, 20 mm loops were necessary in order to support the anterior leaflet, mimicking the increased length of anterior leaflet chordae observed in vivo [6]. Successful repair necessitated only one prosthesis (i.e., ≤3 loops) in 80% of patients (Figure 3). Whilst all patients received an annuloplasty in addition to their neochordae, extra procedures to restore leaflet integrity (e.g., repair of leaflet clefts) were only required in five cases.

Intraoperative echocardiography was utilised for every procedure, and no greater than mild MR was confirmed in all patients at the conclusion of the procedure (Table 4). One of the earlier patients (4th in this series) required a return onto CPB after weaning off, as the Chord-X prosthesis had dislodged from the papillary muscle. The leaflet attachments were all intact; thus, only the papillary muscle attachment needed to be re-anchored. It was reattached to the same papillary muscle head (but slightly deeper) a second time with no further complications, and a durable long-term repair was achieved without the need for re-operation.

Surgical recovery was uneventful for most patients, with a median length of stay of seven days in the hospital. One patient required a return to the theatre for control of bleeding, which resulted in a prolonged stay. Five patients were readmitted within 30 days of operation, either due to arrhythmia or non-cardiac concerns. No readmission was related to heart failure or valve deterioration, and there were no early mortalities.

Postoperative echocardiography data was obtained in all 40 patients at varying time points, and data from the latest follow-up examination is summarized in Table 5. The median time until the latest follow-up echocardiogram was 33 days, with 15 patients having data available greater than three months post-operatively. The longest post-procedure echocardiography follow-up was 1288 days. At the latest follow-up, 75% of patients had nil or trace MR, and only 25% had mild MR. No patient to date has had greater than mild MR or required reintervention to their mitral valve.

## 4. Discussion

Since David first popularised the use of ePTFE neochordae for mitral valve repair [7], various techniques have been suggested for how surgeons should create and implant these neochordae. The most prevalent methods include the use of simple interrupted sutures [8], a continuous running suture [1], or the Leipzig loop technique [9]. No method has been proven to be strictly superior to another, and each method has its own advantages and disadvantages, considering factors such as time taken to construct neochordae, procedural complexity of construction, technical difficulty of achieving correct chordal length, and ease of implantation into the patient. While no in vivo studies compare the efficacy of these techniques, a biomechanical analysis in 2021 demonstrated that each of these configurations can withstand forces far greater than those seen under physiological conditions [10]. Thus, it is up to the surgeon to choose a technique that they can reliably master in order to produce a long-lasting repair.

The Chord-X Pre-Measured Loops system is an iteration of the Leipzig loop technique, whereby multiple chordal loops are pre-created and arise from a single pledget, which will be anchored to a papillary muscle. The primary advantage of this technique is that the loops can be constructed on a back table prior to the initiation of CPB, therefore limiting cross-clamp time [5]. Additionally, all the loops are assembled to be of identical size, ensuring that a consistent repair can be achieved over a wide leaflet area. This is in contrast to the interrupted or continuous suture techniques, where extra technical difficulty is involved in ensuring that each implanted neochord is of the correct length.

Nonetheless, there are several drawbacks of the traditional Leipzig technique, which a premeasured loop system aims to resolve. Firstly, the manually constructed Leipzig loops rely heavily on accurate TOE measurements, as the loops are already constructed prior to intra-operative valve inspection. Secondly, only a certain number of loops are created prior to cross-clamping, and if further loops are needed (due to inaccurate sizing, a misplaced stitch, or a failed repair), then these can be time-consuming to create and contribute to extra ischaemic time. Our experience has shown us that the Chord-X system can circumnavigate these potential weaknesses. Once the mitral valve has been inspected and the appropriate loop size is determined, the Chord-X Pre-Measured Loops can be immediately opened and sutured in place without further configurations. This efficiency proved especially useful, for example, in our patient who required another period of myocardial arrest in order to re-repair the valve. Utilising a system that can be easily implanted or adapted in the case of a failed first repair might influence the decision to re-repair compared to yielding to a valve replacement and thus have long-lasting implications for the patient.

To our knowledge, this study represents the largest case series involving a novel pre-measured chordal loop system and the only study to include post-discharge outcomes. Our most notable finding was that all surgeons at our institution were able to achieve satisfactory mitral valve repair in all cases, as demonstrated by excellent intraoperative echocardiography results and no patient requiring conversion to mitral valve replacement. These repairs proved to be durable after the immediate post-operative period, with no patients having greater than mild MR in their follow-up scans. Additionally, these results were achieved by surgeons with a relatively low annual volume of mitral valve repairs. Large database studies have suggested that low-volume surgeons are at higher risk of conversion to valve replacement [11,12], and thus, a system that mitigates this risk is worth adopting. Significantly, no additional or specialised training was required for surgeons to utilise the Chord-X Pre-Measured Loops system effectively. Each of the surgeon’s first experiences with Chord-X is included in this study, and these patients exhibited comparable results to the rest of the cohort.

We hope that our early success with this system will encourage surgeons to adopt Chord-X Pre-measured Loops or similar novel systems into their mitral repair practice. In the case of single leaflet disease, adequate repair required only one prosthesis to be utilised in almost all patients (Figure 2), highlighting the ease of use and cost-effective nature of such a system. Conversely, in cases of complex bi-leaflet prolapse, a system capable of reliably producing loops of various lengths is invaluable and can be adapted to any patient’s anatomy. Given that our study and that of Gillinov et al. [3] have established short-term safety data of Chord-X Pre-measured Loops, future research should focus on the long-term durability of these systems. Additionally, it might be beneficial to directly compare surgeon experience with pre-measured versus manually constructed loops in terms of ease of repair or bypass times.

## 5. Conclusions

Overall, our results underscore the safety and reproducibility of pre-measured loop systems, particularly the Chord-X Pre-Measured Loops system. Surgeons with relatively lower mitral case volumes were able to implement this system effectively in a low-risk cohort with primarily posterior leaflet prolapse. We acknowledge the study’s limitations, including the small sample size, retrospective design, and relatively short echocardiographic follow-up period. Nevertheless, we hope these findings will encourage surgeons to incorporate these novel systems into their clinical practice. Further adoption of Chord-X Pre-Measured Loops will facilitate future studies comparing this technology with traditional surgeon-constructed chords, providing long-term data.

## Figures and Tables

**Table 1:** Patient Demographics.

	All Patients (*n* = 40)
Age (years)	69 ± 12
Sex (male vs. female)	25 (63%) vs. 15 (37%)
BMI	25.9 ± 4.1
Pre-operative Serum Creatinine	84.6 ± 15.4
Status (*n*, %)	
Elective	37 (92%)
Inpatient	2 (5%)
Emergent	1 (2%)
NYHA Stage (*n*, %)	
1	13 (33%)
2	11 (28%)
3	15 (38%)
4	1 (2%)
Past History (*n*, %)	
Hypertension	12 (30%)
Coronary Artery Disease	8 (20%)
AF	6 (15%)
Diabetes	2 (5%)
CKD	0
Chronic Lung Disease	0
Chronic Liver Disease	0
EuroSCORE II (%)	1.3 ± 0.9

NYHA = New York Heart Association, AF = Atrial Fibrillation, CKD = Chronic Kidney Disease, EuroSCORE = European System for Cardiac Operative Risk Evaluation.

**Table 2:** Pre-operative Echocardiography Characteristics.

	All Patients (*n* = 40)
MV Pathology (*n*, %)	
Degenerative	40 (100%)
Other	0
MR Severity (*n*, %)	
Less than severe	0
Severe	40 (100%)
Mechanism of MR	
Prolapse only	22/40 (55%)
Flail Leaflet	18/40 (45%)
Leaflet(s) Affected (*n*, %)	
P1	3 (8%)
P2	34 (85%)
P3	22 (55%)
Anterior (All of A1,A2,A3)	1 (2%)
EF (%)	57 ± 8
LVEDD (cm) *	5.4 ± 0.7
PASP * (mmHg)	36.1 ± 13.7
MV Gradient (mmHg)	0
TR Severity (*n*, %)	
None or Trivial	15/40 (38%)
Mild	20/40 (50%)
Moderate	5/40 (12%)
AR Severity (*n*, %)	
None or Trivial	32/40 (80%)
Mild	8/40 (20%)

MV = Mitral Valve, MR = Mitral Regurgitation, EF = Ejection Fraction, LVEDD = Left Ventricular End-Diastolic Diameter, PASP = Pulmonary Artery Systolic Pressure, TR = Tricuspid Regurgiutation, AR = Aortic Regurgitation. * Unknown values have been excluded.

**Table 3:** Intra-operative Technique.

	All Patients (*n* = 40)
Approach (MIS vs. Sternotomy)	6 (15%) vs. 34 (85%)
Chord-X Length	
Posterior Leaflet (*n*, %)	
12 mm	6 (15%)
16 mm	34 (85%)
20 mm	0
24 mm	0
Anterior Leaflet (*n*, %)	
12 mm	0
16 mm	0
20 mm	1 (100%)
24 mm	0
Chord-X Papillary Attachments	
Posterior (*n*, %)	37 (93%)
Anterior (*n*, %)	11 (28%)
Chord-X Leaflet Attachments	
P1	3 (8%)
P2	30 (75%)
P3	19 (48%)
Anterior (All of A1, A2, A3)	1 (2%)
C1	0
C2	2 (5%)

MIS = Minimally-invasive Surgery.

**Table 4:** Peri-operative Outcomes.

	All Patients (*n* = 40)
CPB	
Cross-clamp Time (min)	107 ± 33
Total Bypass Time (min)	135 ± 40
Post-op TOE MR	
None or Trivial	35 (88%)
Mild	5 (12%)
Greater than Mild	0
Length of stay (median, IQR)	7 days (6 days–9 days)
Complications	
Readmission	5 (13%)
Return to theatre (bleeding)	1 (3%)
Return to ICU	1 (3%)

CPB = Cardio-pulmonary Bypass, TOE = Trans-oesophageal Echocardiography, MR = Mitral Regurgitation.

**Table 5:** Follow-up Echocardiography.

	All Patients (*n* = 40)
Time until follow-up TTE (median, IQR)	33 days (6 days–187 days)
Follow-up MR (*n*, %)	
None or Trivial	30 (75%)
Mild	10 (25%)
**Greater than Mild**	**0**
Follow-up EF (%)	51 ± 7
Follow-up LVEDD * (cm)	4.7 ± 0.7
Decrease in LVEDD * (cm)	0.7 ± 0.7
Follow-up PASP * (mmHg)	29.2 ± 9.0
Decrease in PASP (mmHg)	8.4 ± 15.8

TTE = Trans-thoracic Echocardiography, MR = Mitral Regurgitation, EF = Ejection Fraction, LVEDD = Left Ventricular End-Diastolic Diameter, PASP = Pulmonary Artery Systolic Pressure. * Unknown values have been excluded.

## Data Availability

Data is contained within the article or Supplementary Materials.

## Supplementary Materials

The following supporting information can be downloaded at: https://www.mdpi.com/article/10.3390/jcm13237029/s1, CSV S1: De-identified Data
